# Supplementary figures and images for: Blood pressure and bladder cancer risk in men by use of survival analysis and in interaction with NAT2 genotype, and by Mendelian randomization analysis
Source: PLoS One. 2020 Nov 25;15(11):e0241711. doi: 10.1371/journal.pone.0241711 (PMC7688142; doi:10.1371/journal.pone.0241711)

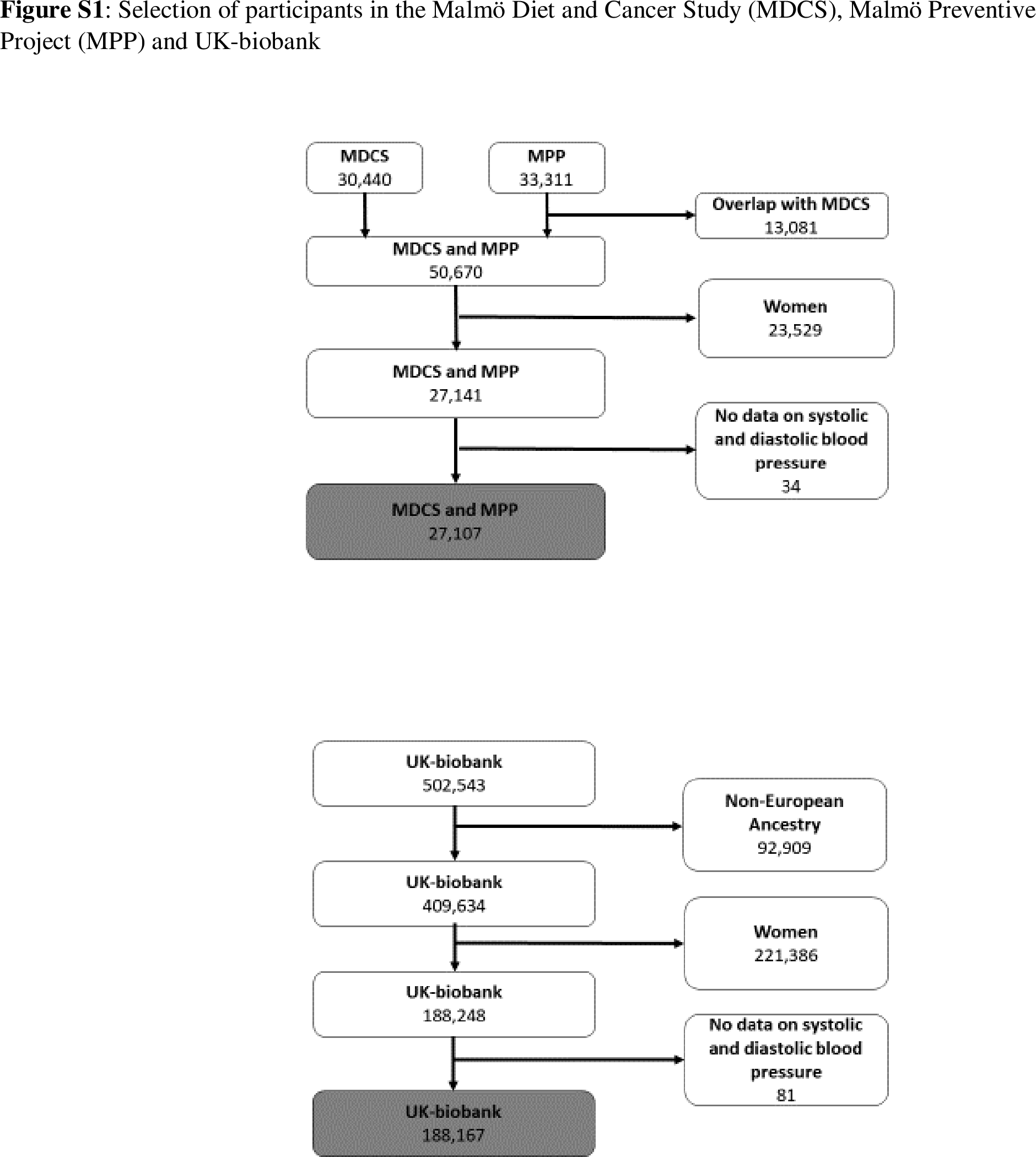

Supplement: S1 Fig — (TIF) [file pone.0241711.s003.tif]

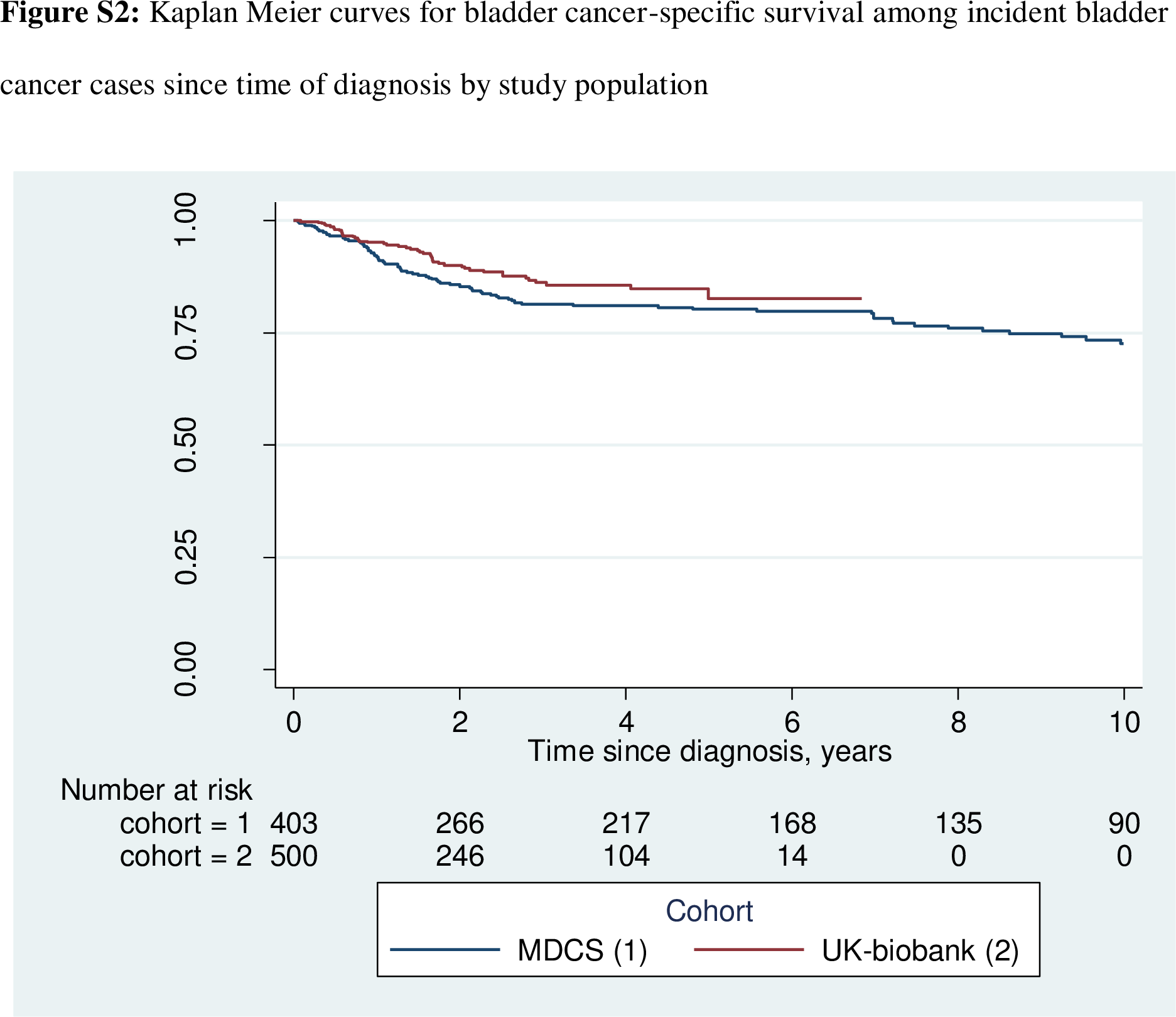

Supplement: S2 Fig — (TIF) [file pone.0241711.s004.tif]

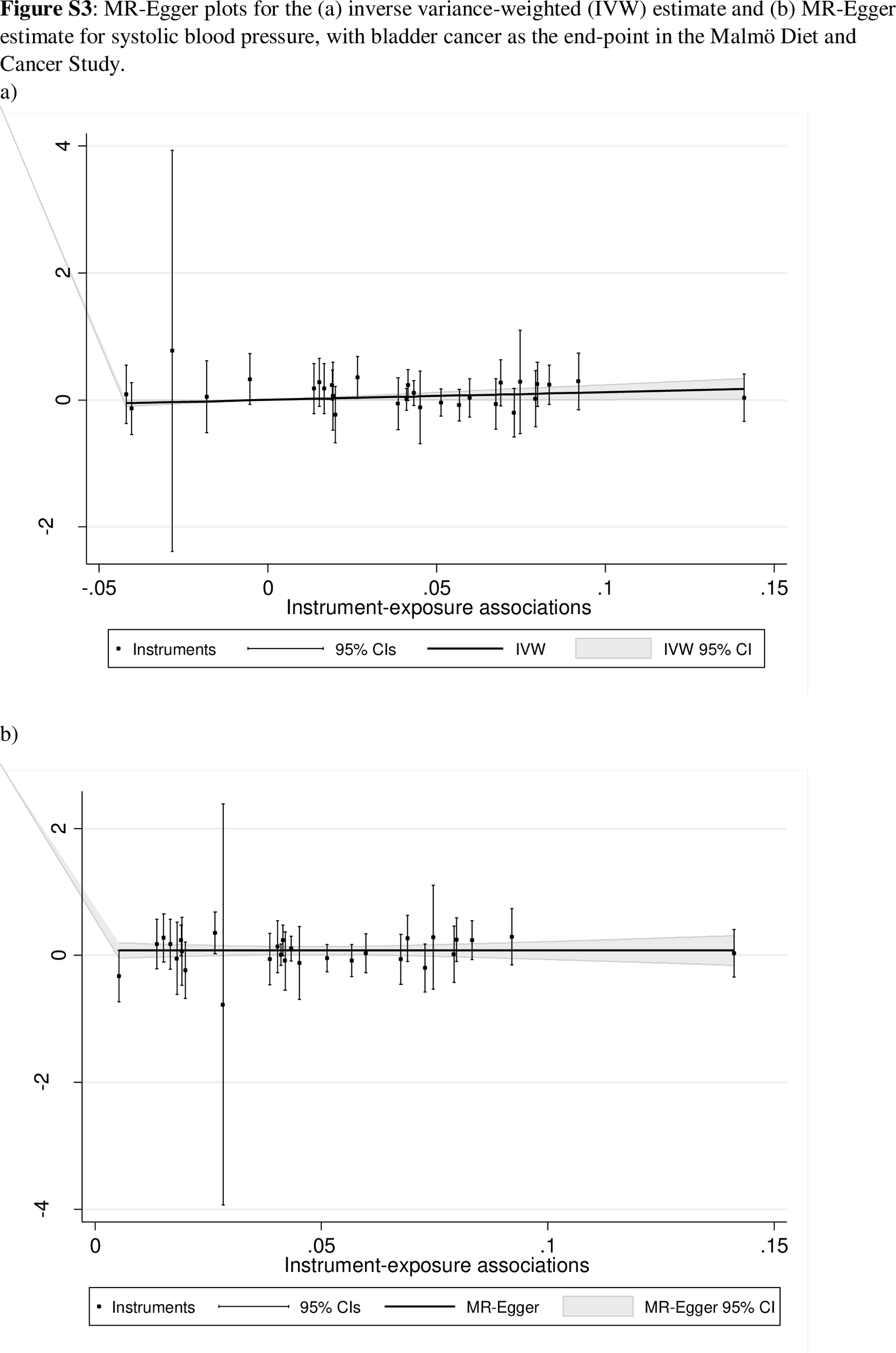

Supplement: S3 Fig — (TIF) [file pone.0241711.s005.tif]

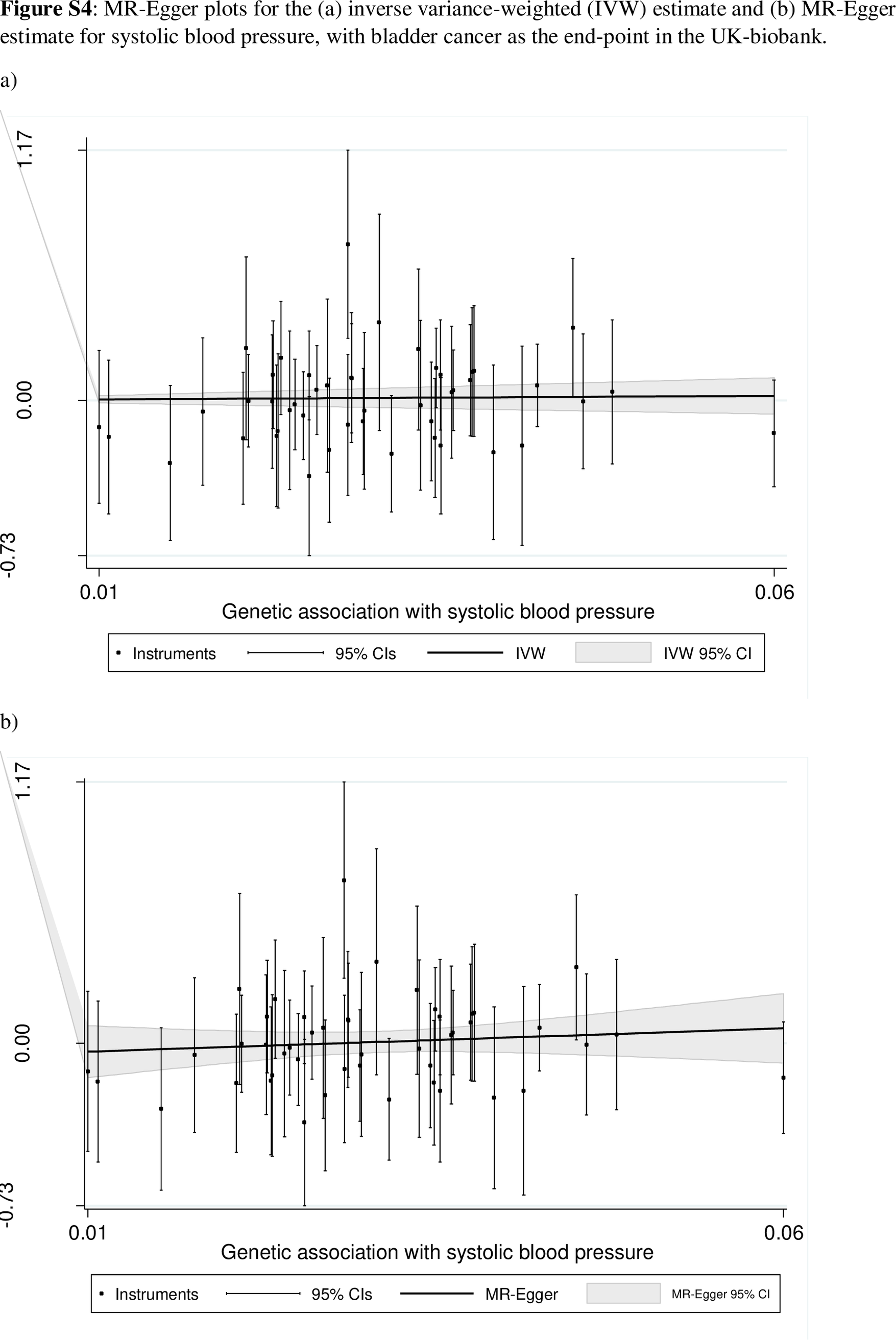

Supplement: S4 Fig — (TIF) [file pone.0241711.s006.tif]

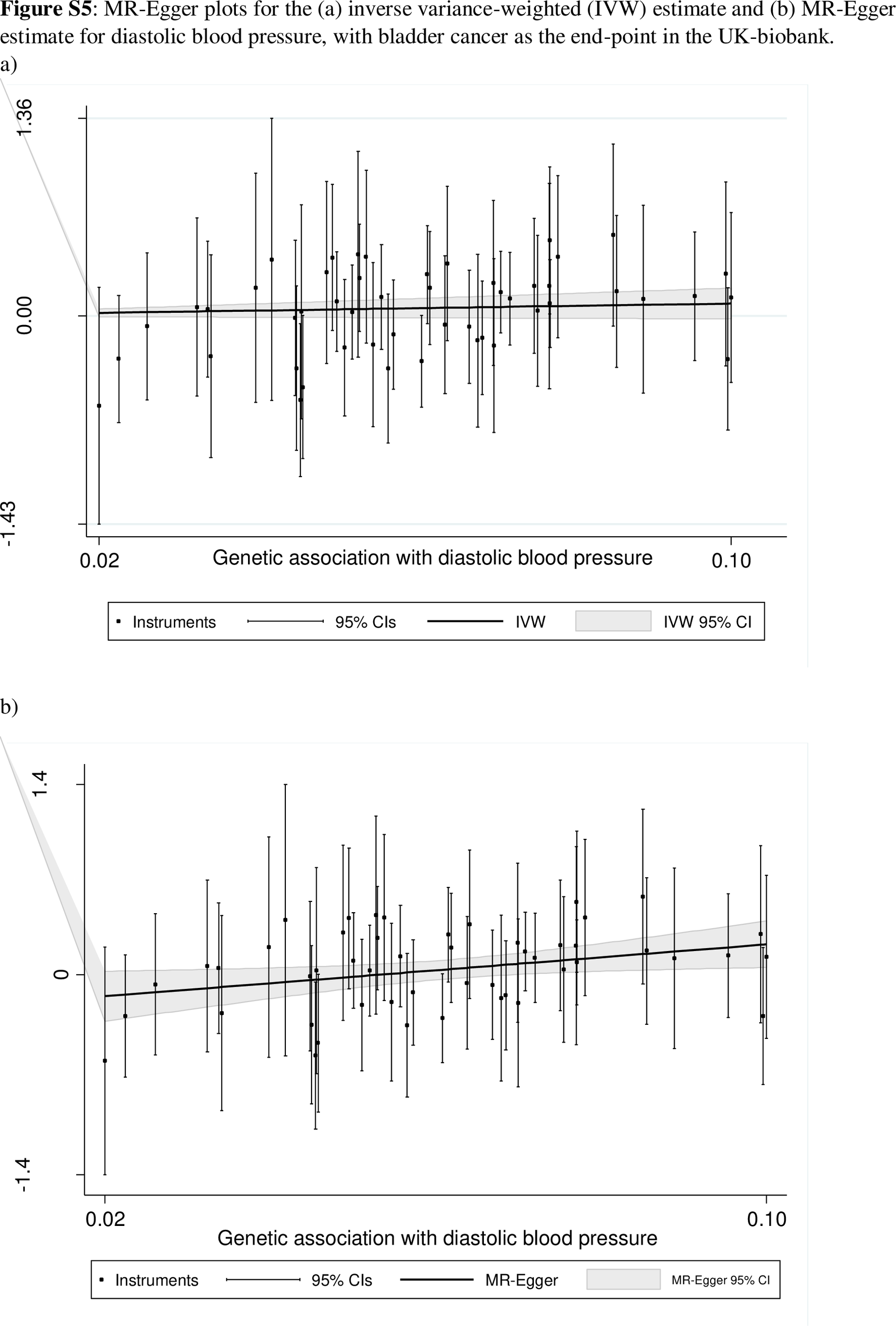

Supplement: S5 Fig — (TIF) [file pone.0241711.s007.tif]

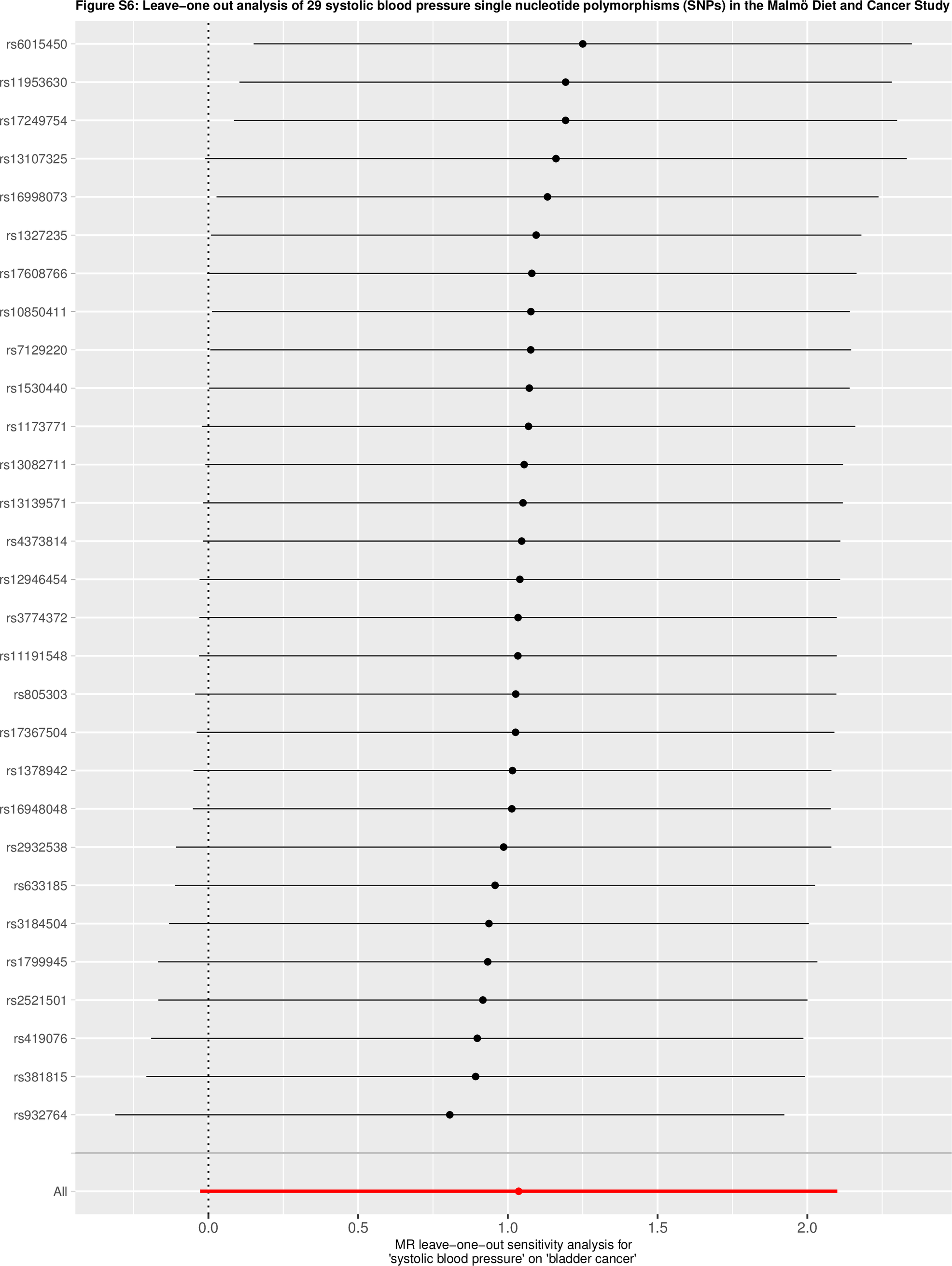

Supplement: S6 Fig — (TIF) [file pone.0241711.s008.tif]

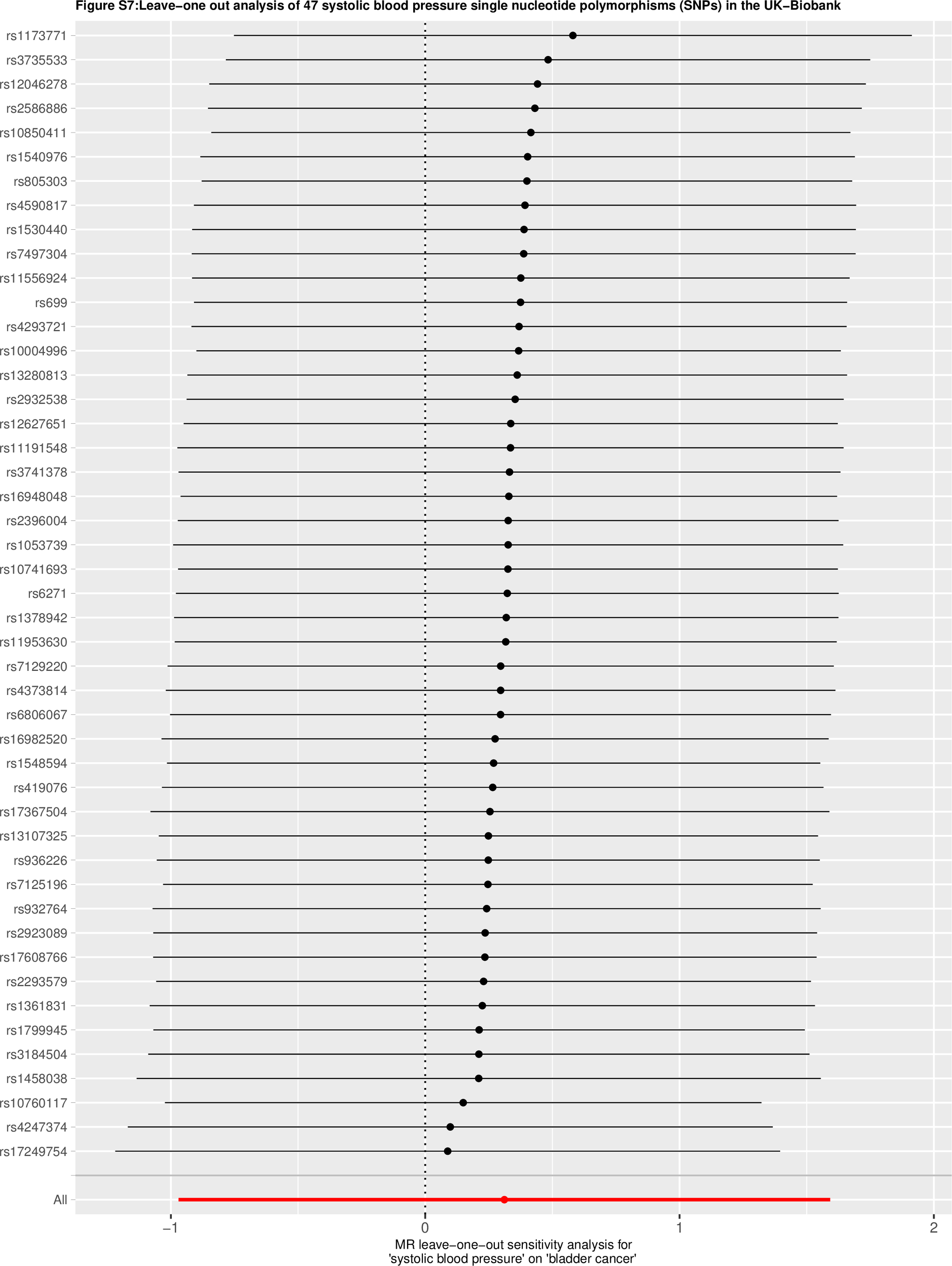

Supplement: S7 Fig — (TIF) [file pone.0241711.s009.tif]

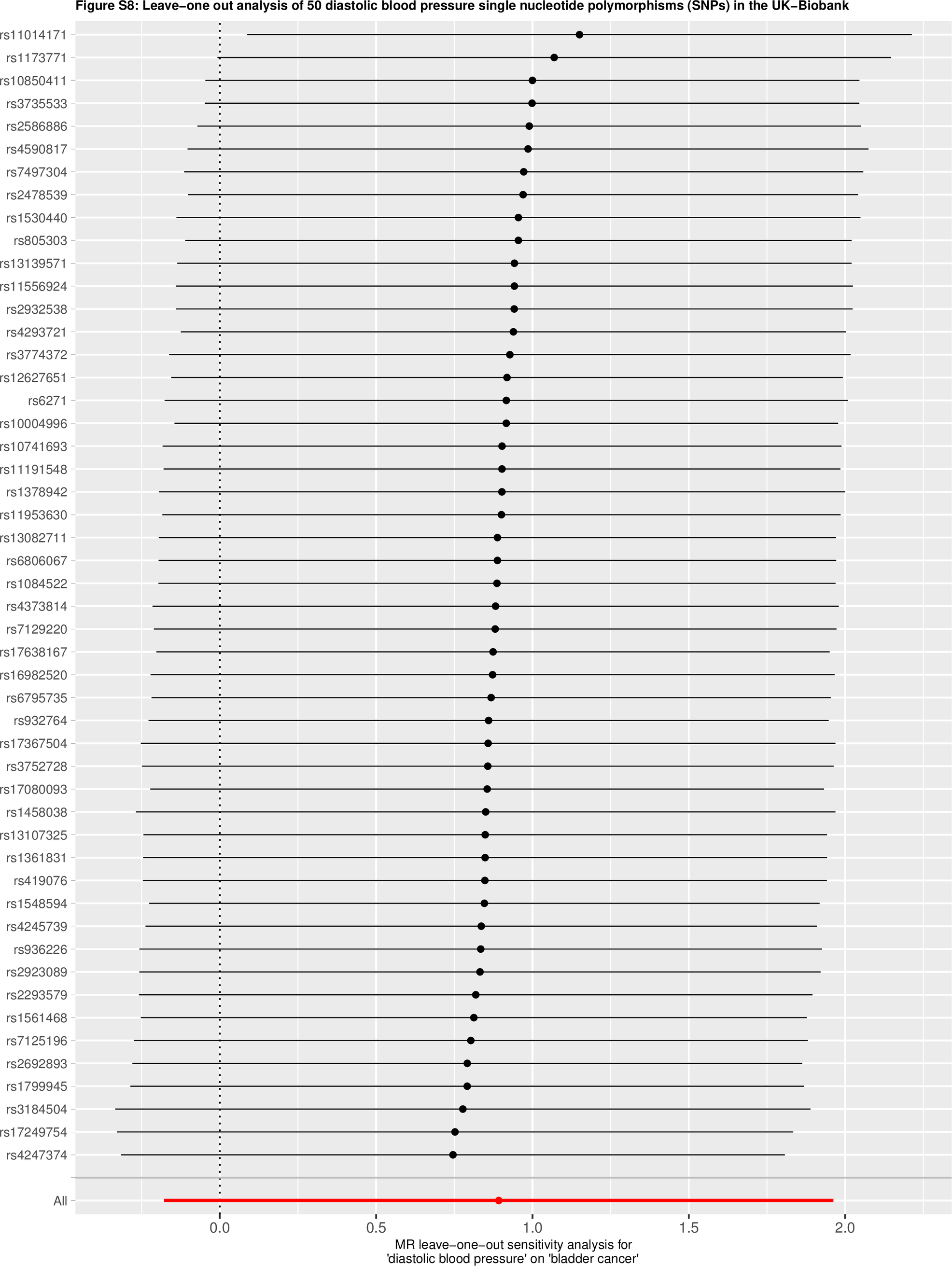

Supplement: S8 Fig — (TIF) [file pone.0241711.s010.tif]
