# Supplementary material for: Blood pressure and bladder cancer risk in men by use of survival analysis and in interaction with NAT2 genotype, and by Mendelian randomization analysis
Source: PLoS One. 2020 Nov 25;15(11):e0241711. doi: 10.1371/journal.pone.0241711 (PMC7688142; doi:10.1371/journal.pone.0241711)
Supplement: S1 Table — (PDF) [file pone.0241711.s011.pdf]

S1 Table : Baseline characteristics women in the Swedish cohorts and the UK-biobank

Characteristic	MDCS and MPP (n=23,484)	UK-biobank (n=221,114)
**Baseline year, range**	1974-1996	2006-2010
**Baseline age, years, mean (SD)**	55.5 (8.7)	57.2 (7.9)
**Category, n (%)**		
<30	411 (1.8)	0 (0.0)
30-44	1,010 (4.3)	20,609 (9.2)
45-59	14,968 (63.7)	103,447 (46.9)
≥60	7,095 (30.2)	97,058 (43.9)
**Smoking status, n (%)***		
Never smoker	9,931 (44.2)	130,889 (59.4)
Ex-smoker	5,537 (24.7)	70,339 (31.9)
Current smoker	6,989 (31.1)	19,126 (8.7)
**Pack years among current smokers, n (%)***		
<10	1,825 (26.3)	2,492 (15.3)
10-19.9	1,521 (21.9)	4,013 (24.8)
≥20	3,606 (51.8)	9,710 (59.9)
**Blood pressure, mm Hg, mean (SD)**		
Systolic blood pressure	136.2 (20.4)	137.6 (20.2)
Diastolic blood pressure	83.6 (9.7)	80.7 (10.5)
**Category, systolic/diastolic, n (%)**		
<140/90 mm Hg	12,023 (51.2)	120,109 (54.3)
140/90-159/99 mm Hg	7262 (30.9)	66,059 (29.9)
≥160/100 mm Hg	4,199 (17.9)	34,946 (15.8)
**BMI, kg/m^2, mean (SD)†**		
<18.5	443 (1.9)	1,627 (0.7)
18.5-24.9	12,305 (52.5)	86,149 (39.1)
25-29.9	7,531 (32.1)	81,613 (37.0)
≥30	3,183 (13.5)	51,098 (23.2)
**Mean follow-up time, years (SD)**	20.5 (7.8)	5.7 (1.2)
**Follow-up time, n (%)**		

<5	2,523 (10.7)	66,739 (30.2)
5-9	1,465 (6.2)	154,375 (69.8)
10-14	1,930 (8.3)	0 (0.0)
≥15	17,566 (74.8)	0 (0.0)

[*] Smoking status was missing for 760 (0.3%) women in the UK-biobank and for 1,027 (4.4%) women in the MDCS and MPP combined. Accumulated pack-years among current smokers, excluding 38 (0.5%) and 2,911 (1.3%) current smokers with missing pack-years data in the MPP and MDC combined and UK-biobank respectively.

[†] BMI data was missing for 627 women in the UK-biobank and 22 women in MDCS and MPP combined.
