# Supplementary material for: Blood pressure and bladder cancer risk in men by use of survival analysis and in interaction with NAT2 genotype, and by Mendelian randomization analysis
Source: PLoS One. 2020 Nov 25;15(11):e0241711. doi: 10.1371/journal.pone.0241711 (PMC7688142; doi:10.1371/journal.pone.0241711)
Supplement: S2 Table — Abbreviations: SBP, systolic blood pressure; DBP, diastolic blood pressure; IV, instrumental variable; MDCS, Malmö diet and Cancer Study; OR, odds ratio; BMI, body mass index. a For age at baseline, date of birth, BMI, smoking, and education, we used linear regressions to investigate the association with blood pressure indices and their respective genetic scores. For physical activity and antihypertensive medication, we used logistic regression. (PDF) [file pone.0241711.s012.pdf]

S2 Table . Association between per standard deviation of measured and instrumental variables of systolic and diastolic blood pressure, and potential confounders in the relationship between blood pressure and bladder cancer risk

**Potential confounders** [a]	**MDCS**		**UK Biobank**			
	**SBP IV** **β/OR (p-value)**	**SBP** **β/OR (p-value)**	**SBP IV** **β/OR (p-value)**	**SBP** **β/OR (p-value)**	**DBP IV** **β/OR (p-value)**	**DBP** **β/OR (p-value)**
**Age at baseline**	0.08 (0.722)	0.42 (<0.001)	0.01 (0.960)	0.17 (<0.001)	0.07 (0.258)	-0.04 (<0.001)
**Date of birth**	-0.08 (0.698)	-0.42 (<0.001)	-0.02 (0.750)	-0.22 (<0.001)	-0.11 (0.163)	0.04 (<0.001)
**BMI**	-0.82 (0.153)	0.23 (<0.001)	-0.21 (<0.001)	0.22 (<0.001)	-0.60 (<0.001)	0.32 (<0.001)
**Smoking**	-0.21 (0.427)	-0.05 (0.002)	0.01 (0.721)	-0.01 (<0.001)	0.03 (0.622)	-0.02 (<0.001)
**Education**	-0.66 (0.041)	-5.16 (0.036)				
**Physical activity**	-0.10 (0.759)	0.07 (<0.001)				
**Antihypertensive medication**	2.95 (<0.001)	0.61 (<0.001)				

Abbreviations: SBP, systolic blood pressure; DBP, diastolic blood pressure; IV, instrumental variable; MDCS, Malmö diet and Cancer Study; OR, odds ratio; BMI, body mass index.

[a] For age at baseline, date of birth, BMI, smoking, and education, we used linear regressions to investigate the association with blood pressure indices and their respective genetic scores. For physical activity and antihypertensive medication, we used logistic regression.
