# Supplementary material for: Blood pressure and bladder cancer risk in men by use of survival analysis and in interaction with NAT2 genotype, and by Mendelian randomization analysis
Source: PLoS One. 2020 Nov 25;15(11):e0241711. doi: 10.1371/journal.pone.0241711 (PMC7688142; doi:10.1371/journal.pone.0241711)
Supplement: S3 Table — (PDF) [file pone.0241711.s013.pdf]

S3 Table : Odds ratio (95% confidence interval) from Mendelian randomization analysis of incident bladder cancer, and incident and prevalent bladder cancers combined, for systolic and diastolic blood pressure in the Malmö Diet and Cancer Study and UK-biobank.

	**UK-biobank**					**Malmö Diet and Cancer Study**			
		**Incident cases only ($N_{individuals}$=188,167)**		**Incident and prevalent cases ($N_{individuals}$=188,167)**			**Incident cases only ($N_{individuals}$=11218)**		**Incident and prevalent cases ($N_{individuals}$=11218)**
		**N_{cases}=502**		**N_{cases}=1014**		**N_{cases}=367**		**N_{cases}=411**	
**Exposure**	**Analysis**	**R^2[a]**	**OR (95% CI)**	**R^2**	**OR (95% CI)**	**R^2**	**OR (95% CI)**	**R^2**	**OR (95% CI)**
**Systolic BP**	**2SLS**	0.5%	1.24 (0.35-4.40)	0.5%	1.18 (0.48-2.87)	0.61%	7.70(1.92-30.9)	0.61%	5.50 (1.47-20.5)
	**IVW**		1.37 (0.43-4.37)		1.13 (0.50-2.57)		3.43 (1.12-10.5)		2.82 (0.97-8.17)
		**Incident cases only ($N_{individuals}$=188,167)**		**Incident and prevalent cases ($N_{individuals}$=188,167)**					
		**N_{cases}=498**			**N_{cases}=1008**				
**Diastolic BP**	**2SLS**	0.7%	1.97 (0.68-6.14)	0.7%	1.44 (0.65-3.19)				
	**IVW**		2.47 (0.87-7.02)		1.42 (0.68-2.96)				

Abbreviation: OR, odds ratio; BP, blood pressure; IVW, inverse variance weighted

[a] R^2 is the proportion of the variance in blood pressure explained by the genetic score
