# Supplementary material for: Blood pressure and bladder cancer risk in men by use of survival analysis and in interaction with NAT2 genotype, and by Mendelian randomization analysis
Source: PLoS One. 2020 Nov 25;15(11):e0241711. doi: 10.1371/journal.pone.0241711 (PMC7688142; doi:10.1371/journal.pone.0241711)
Supplement: S4 Table — Abbreviations: MDCS, Malmö Diet and Cancer Study; OR, odd ratio; CI, confidence intervals; BP, blood pressure; 2SLS, two-stage least square regression; IVW, inverse-variance weighted. a R2 is the proportion of BP variance that is explained the genetic score. (PDF) [file pone.0241711.s014.pdf]

S4 Table : Two stage least square regression and inverse variance weighted method for systolic and diastolic blood pressure in relation to bladder cancer incidence for men and women combined in the Malmö Diet and Cancer Study and UK-biobank

		**MDCS ($N_{individuals}$=28264)**		**UK-biobank ($N_{individuals}$=400870)**	
		**N_{cases}=514**		**N_{cases}=671**	
**Exposure**	**Analysis**	**R^{2a}**	**OR (95%CI)**	**R^2**	**OR (95%CI)**
**Systolic BP**	**2SLS**	0.6%	4.43 (1.44-13.7)	0.61%	0.81 (0.31-2.13)
	**IVW**		4.37 (0.94-20.3)		0.75 (0.30-1.85)
**Diastolic BP**	**2SLS**			0.7%	1.33 (0.53-3.32)
	**IVW**				1.38 (0.59-3.23)

Abbreviations: MDCS, Malmö Diet and Cancer Study; OR, odd ratio; CI, confidence intervals; BP, blood pressure; 2SLS, two-stage least square regression; IVW, inverse-variance weighted.

[a] R^2 is the proportion of BP variance that is explained the genetic score
